# Neurometabolomic impacts of wood smoke and protective benefits of anti-aging therapeutics in aged female C57BL/6J mice

**DOI:** 10.21203/rs.3.rs-5936676/v1

**Published:** 2025-03-17

**Authors:** David Scieszka, Jonathan Hulse, Haiwei Gu, Amanda Barkley-Levenson, Ed Barr, Marcus Garcia, Jessica G Begay, Guy Herbert, Mark McCormick, Jonathan Brigman, Andrew Ottens, Barry Bleske, Kiran Bhaskar, Matthew J Campen

**Affiliations:** University of New Mexico College of Pharmacy; University of New Mexico Health Sciences Center; Arizona State University; University of New Mexico College of Pharmacy; University of New Mexico College of Pharmacy; University of New Mexico College of Pharmacy; University of New Mexico College of Pharmacy; University of New Mexico College of Pharmacy; University of New Mexico Health Sciences Center; University of New Mexico Health Sciences Center; Virginia Commonwealth University; University of New Mexico College of Pharmacy; University of New Mexico Health Sciences Center; University of New Mexico College of Pharmacy

**Keywords:** Particulate matter, wildfire smoke, metabolomics, brain, depression

## Abstract

**Background:**

Wildland fires have become progressively more extensive over the past 30 years in the United States, routinely generating smoke that deteriorates air quality for most of the country. We explored the neurometabolomic impact of biomass-derived smoke on older (18 months) female C57BL/6J mice, both acutely and after 10 weeks of recovery from exposures.

**Methods:**

Mice were exposed to wood smoke (WS) 4 hours/day, every other day, for 2 weeks (7 exposures total) to an average concentration of 448 μg particulate matter (PM)/m^3^ per exposure. One group was euthanized 24 hours after the last exposure. Other groups were then placed on 1 of 4 treatment regimens for 10 weeks after wood smoke exposures: vehicle; resveratrol in chow plus nicotinamide mononucleotide in water (RNMN); senolytics via gavage (dasatanib + quercetin; DQ); or both RNMN with DQ (RNDQ).

**Results:**

Among the findings, the aging from 18 months to 21 months was associated with the greatest metabolic shift, including changes in nicotinamide metabolism, with WS exposure effects that were relatively modest. WS caused a reduction in NAD + within the prefrontal cortex immediately after exposure and a long-term reduction in serotonin that persisted for 10 weeks. The serotonin reductions were corroborated by behavioral changes, including increased immobility in a forced swim test, and neuroinflammatory markers that persisted for 10 weeks. RNMN had the most beneficial effects after WS exposure, while RNDQ caused markers of brain aging to be upregulated within WS-exposed mice.

**Discussion:**

Taken together, these findings highlight the persistent neurometabolomic and behavioral effects of woodsmoke exposure in an aged mouse model. Further examination is necessary to determine the age-specific and species-determinant response pathways and duration before complete resolution occurs.

## INTRODUCTION

Over the past 30 years, there has been a documented increase in the extent of wildfires in the United States, which release airborne particulate matter[1] and gaseous pollutants that impact populations far from the fire source.[2–5] In humans, Alzheimer’s disease related dementias (ADRD)[6, 7], suicide[6, 8], depression[6, 9], psychosis[6, 10], and other neurological outcomes have been associated with particulate matter (PM) irrespective of sources[11–13], and specifically from wildfire smoke[14]. Recent epidemiological[14, 15] and preclinical animal[16, 17] studies document potential neurological outcomes of exposure to wildfire smoke, which impacts much of the global population. As the severity of the fires appears to arise from global climate change and increased aridity, identifying individual strategies for protection may be an important public health intervention. Pharmacological interventions hold promise for cognitive benefits and cardioprotective effects, which could offset wildfire smoke-induced neurological and cardiovascular conditions. Wildfire smoke has been associated with pulmonary senescence fates[18] as well as adverse neurological outcomes arising from inflammation[16, 19]. Senescence is a proinflammatory state of cell cycle arrest that has been hypothesized as a root cause of aging[20–23], and can cause systemic inflammation through the release of senescence-associated secretory phenotype molecules into the circulation[24]. To combat this release, senolytics, such as dasatinib and quercetin, are a class of naturally occurring drugs that target and block senescent cell anti-apoptotic pathways, allowing for apoptosis to occur[25].

Resveratrol is a natural polyphenol that has shown effects as an anti-inflammatory[26, 27], cancer management[27, 28], neuroprotective[26, 27], and cardioprotective molecule[27, 29, 30], with many of these effects arising from the activation of sirtuin 1 and other nicotinamide adenine dinucleotide (NAD^+^)-consuming enzymes. Nicotinamide mononucleotide (NMN) is a NAD^+^ precursor[31], whose supplementation has been shown as beneficial for cellular NAD^+^ abundance, longevity[32], cognition[33], ADRD[34], and depression[35], among others[36]. Many investigations have reported antioxidant effects from supplementation with resveratrol[37, 38] and nicotinamide mononucleotide (NMN)[39, 40, 40], and healthspan benefits from the combination of senolytics dasatinib + quercetin (DQ)[41–43]. However, only recently have studies been performed on the combination of resveratrol with NMN (RNMN)[44], and no studies have combined resveratrol, NMN, dasatinib, and quercetin (RNDQ). Therefore, we examined whether the combinations of RNMN, and the more complete cocktail of RNDQ would elicit additive benefits in terms of diminishing negative impacts of laboratory-generated wood smoke (WS) inhalation on neurological health, and possibly outcomes simply related to advanced aging.

For the current study, metabolomic changes in the prefrontal cortex were queried based on its known role in cognition and in neurodegenerative diseases, both of which have been implicated in population level studies of wildfire smoke [14, 38]. The study was designed not only to explore potential persistent effects of WS after cessation of exposure, but also to explore how WS exposure to alter the trajectory of aging across a period of decline, from 18 to 21 months of age.

## RESULTS

### Exposure conditions: PM concentration and size distribution

To examine neurometabolomic effects of wildfire smoke, whole-body inhalation exposures to WS or filtered air (FA) were conducted for 4 h every other day for 14 days (7 total exposures). Female 18-month-old mice were exposed to an average concentration per exposure of 448 μg/m^3^ for each of the 4h exposures (Supplemental Fig. 1), with a 24-hour exposure concentration (in line with U.S. Environmental Protection Agency regulatory methods) of 37 μg/m^3^. Carbon monoxide and oxides of nitrogen were also monitored to reflect the gaseous component of biomass combustion emissions, and these averaged 3.2 ppm and 4ppb, respectively (Supplemental Fig. 1). Carbon monoxide and oxides of nitrogen species were well below EPA National Ambient Air Quality Standard levels. PM size distribution was acquired without mice in the exposure chamber, and distribution was measured over a single 2-hour run. Particle size distribution largely fell within PM _0–1_ (median range = 0.138–0.145 μm) with less than 1% of particles above PM_2.5_. WS exposures did not impact body weight trends in any of the drug regimens, compared to FA (Supplemental Fig. 3).

### Prefrontal Cortex (PFC) Untargeted Metabolomic Effects of Biomass Smoke

**Figure 1:**
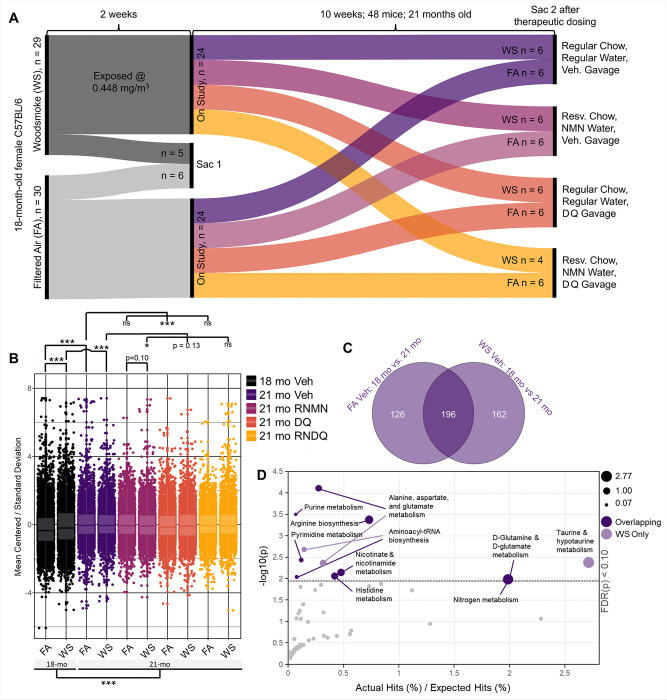
Schematic of experimental design and initial difference testing and metabolomic aging verification. A) Experimental design schematic. 18-month-old female C57BL/6J mice were exposed to WS at an average concentration of 448 μg/m^3^ every other day for 2 weeks (7 total exposures). 1 day post exposures, one cohort of FA and WS were euthanized. The remaining mice went on study for 10 weeks in their individual drug groups. At the end of therapeutic dosing, each cohort was euthanized for metabolomic sequencing. B) Linear mixed effects regression modeling the entire metabolomic dataset. C) Venn diagram of statistically significant metabolites. (student’s t-tests). D) Significant pathways affected by metabolites that overlapped (dark purple) and those that were exclusive to WS (light purple). KEGG pathways for *Mus musculus*.

To assess the long-term persistence of neurometabolomic effects and potential protection from resveratrol, NMN, and senolytics, after the last exposure, two groups of mice were euthanized (6 FA and 5 WS-exposed mice) while the remaining 48 mice were then randomized to 1 of the 4 drug regimens for 10 weeks after exposures (Fig. 1A): (1) standard chow, deionized (DI) water, vehicle (Veh) gavage; (2) resveratrol milled into standard chow, NMN water, Veh gavage (RNMN); (3) standard chow, DI water, dasatinib and quercetin (DQ) gavage; (4) resveratrol chow, NMN water, DQ gavage (RNDQ). Of the 40 total 18-month-old mice exposed to WS, only one died during the 14d exposure period, while no FA controls died. In the subsequent 10 weeks of pharmacological treatment, 2 of the WS mice (out of 34) died before study completion, both in the RNDQ treatment arm; no FA mice died prematurely.

There were significant metabolomic differences between 18-month-old FA and WS in the PFC, indicating an exposure effect (Fig. 1B). The natural course of aging also significantly altered PFC metabolites, through the comparisons of 18mo Veh to 21mo Veh for both FA and WS, and through the grouped comparison of FA&WS 18mo to FA&WS 21mo (also illustrated by the principal component analysis, Supplemental Fig. 4). The RNMN intervention group trended differently between 21mo FA vs WS exposure conditions and was significantly different between exposure-matched WS 21mo Veh. FA RNMN vs FA Veh showed no differences. However, the FA DQ group was significantly different compared to age-matched FA Veh. These findings indicated that the combination of DQ had the strongest effect on the overall PFC metabolic profile of unexposed mice, while RNMN had the strongest effect on WS-exposed mice.

#### Aging Influence on PFC Metabolites:

To interrogate the natural aging effect further, significant metabolite differences from 18 months to 21 months, for both FA and WS groups, were compiled into lists and compared as a Venn diagram (Fig. 1C). We observed a greater number of significant overlapping metabolites (196) than significant non-overlapping metabolites in FA (126) or WS (162). The overlapping cluster reflects aging-related changes that are unperturbed by WS exposure. We extracted the significant overlapping metabolites and input them into pathway analysis. After FDR correction (cut-off p < 0.1), the main pathways affected included alanine, aspartate, and glutamate metabolism; purine biosynthesis; arginine biosynthesis; pyrimidine metabolism; and nicotinate and nicotinamide metabolism (Fig. 1D, Supplemental Table 1). The affected pathway of nicotinate and nicotinamide metabolism confirms the accuracy of pathway analysis employed, based on abundant literature showing that aging is associated with declines in NAD^+^ reserves and biosynthesis[45–55]. We then took the non-overlapping WS metabolites and queried them for pathway alterations. Previous work from our lab showed that wildfire exposures decreased neuroprotective taurine[16], which was confirmed here as one of the main affected pathways: taurine and hypotaurine metabolism. Taken together, these results indicate that the effect of aging over this 10-week period in mice has a greater impact on the metabolic profile than the effects of our subchronic WS exposure. Additionally, aging had a greater impact than any drug combination. However, the modest WS exposure levels still affected the metabolic profile after 10 weeks of natural recovery; and the drug combinations most affecting PFC’s of FA and WS are DQ and RNMN, respectively.

#### WS and Drug Interventions Effects on Metabolites:

**Figure 2:**
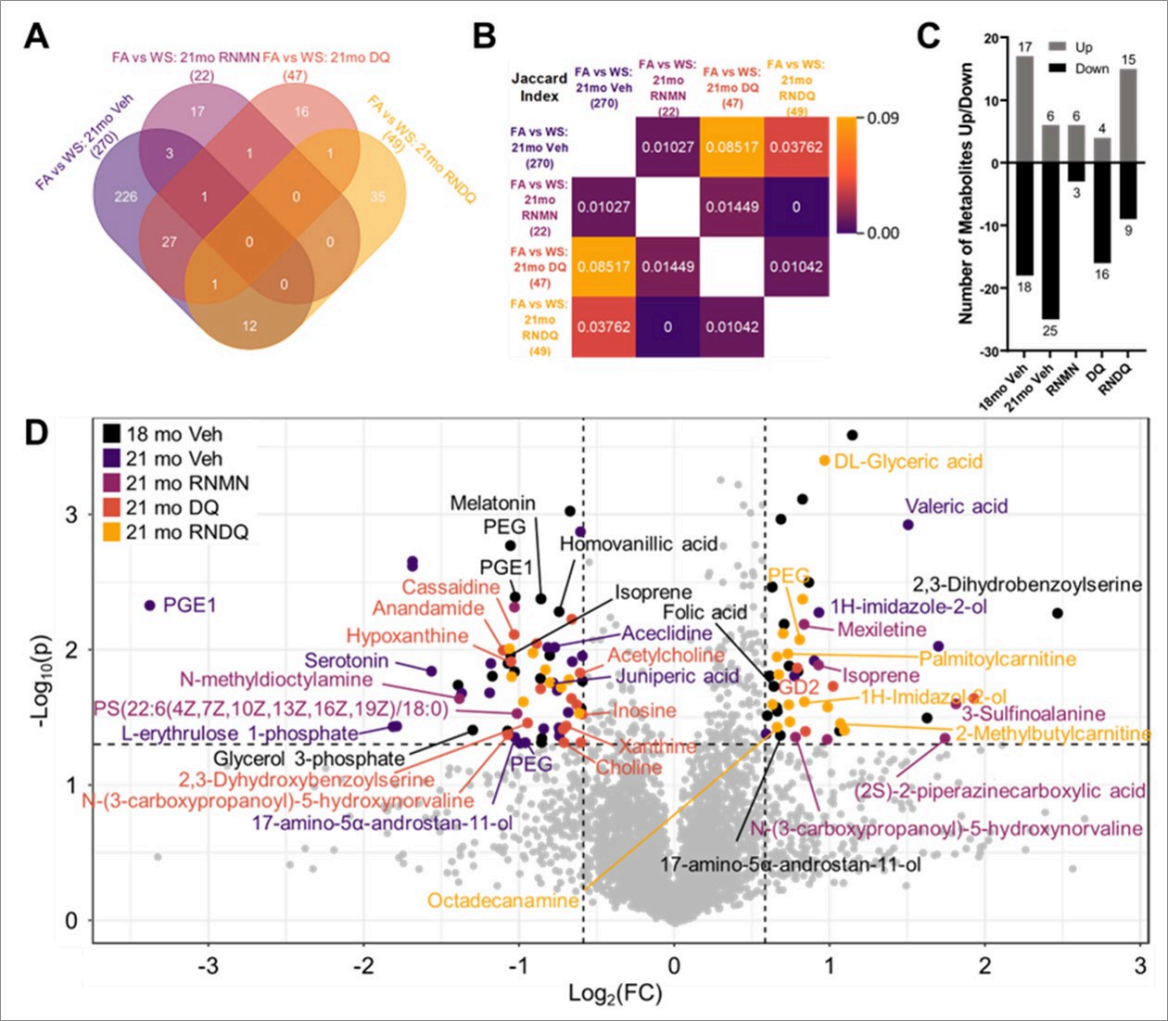
Drug correlations and overlay of significantly altered metabolites. A) Venn diagram of statistically significant metabolites. (student’s t-tests). B) For each group, Jaccard indices were calculated C) Significant volcano-plot-calculated metabolites up/down per condition comparing FA versus WS. D) Overlay of untargeted and NAD+ metabolomic panels. In order from top to bottom, Black: young, 18mo-old mice. Dark purple: natural course of aging for 10 weeks after WS exposure without drug intervention. Light purple: resveratrol and NMN. Orange: dasatinib and quercetin (DQ). Yellow: RNMN + DQ (RNDQ).

We then sought to determine the WS and drug intervention impact on metabolic profile and asked which drug combination would have the weakest overlap with the comparison between FA vs WS: 21 mo Veh (Fig. 2). The comparison of FA vs WS at 21mo timepoint should reveal the metabolic profile skewed by WS, rather than the natural effects of aging. A weak overlap between a drug combination with the WS profile could indicate a metabolic shift away from the deleterious exposure outcomes. The comparisons examined FA 21mo vs WS 21mo mice, with significant metabolites totaling 270 for Veh, 22 for RNMN, 47 for DQ, and 49 for RNDQ. Venn diagrams revealed the weakest overlap between Veh and RNMN (3 exclusive + 1 shared with DQ), middle overlap of Veh with RNDQ (13), and strongest overlap between Veh and DQ (29; Fig. 2B). However, these overlaps were relatively small compared to the overall outcomes of FA vs WS Veh at the 21mo timepoint (270). Relative to the other conditions, this indicated that DQ was least able to shift metabolic profile away from the WS exposure profile, with the combination of RNMN having the greatest shift after WS exposure. We also employed a Jaccard calculation to remove weighted bias based on the total number of significantly altered metabolites per condition. Regardless of methodology employed, we see the strongest overlap with Veh and DQ (J_index_: 0.08517), a middle overlap of Veh with RNDQ (0.0376), and the weakest overlap of Veh with RNMN (0.0103; Fig. 2B). These indicated a minor reduction in effectiveness of drug combinations DQ and RNDQ after WS exposure and prompted a thorough investigation into individual metabolite contributions.

Volcano plots were compiled for drug-age-matched exposure condition (FA vs WS). The number of significantly up and downregulated metabolites revealed several patterns (Fig. 2C). Both ages of (FA vs WS) Veh had the largest number of metabolites that were significantly *decreased* (18mo: 18; 21mo: 25), while 18mo Veh and RNDQ had the greatest number that were significantly *increased* (18mo: 17; RNDQ:15; Fig. 2C). Of the drugs, RNDQ had the largest total of significantly altered metabolites in the FA vs WS condition (24) while RNMN had the least number of significantly altered (9). Together, these indicated an inability to fully resolve WS exposure over 10 weeks without intervention, and that RNMN most effectively resolved WS changes while RNDQ elicited the greatest differential drug response between FA and WS. Taken together, these data illustrate the differences seen at each level of examination (with and without corrections). Without FDR correction, Venn diagrams revealed broad metabolic profiles that explained the aging effect (Figs. 1C; 2A, B). With FDR correction, pathway analyses, lmer, and volcano plots revealed the metabolites most responsible for metabolic shifts (Figs. 1B, D; 2D).

Overall, the volcano plot for 18mo Veh illustrated a WS effect that is larger than expected from such a modest exposure (Fig. 2D). After 10 weeks, the 21mo Veh mice still had not fully resolved this response to baseline. Of the drug combinations, RNDQ caused the upregulation of two metabolites known to exist within the aging murine brain (DL-glyceric acid[56] and octadecylamine[57]). This indicates a potential hazardous outcome for taking this drug regimen after WS exposure. Finally, RNMN appeared to recover the PFC to a greater extent than no intervention (21mo Veh), DQ on its own, and the combination of RNDQ (Fig. 2C, D).

### Targeted PFC Pathway Metabolomics

**Figure 3:**
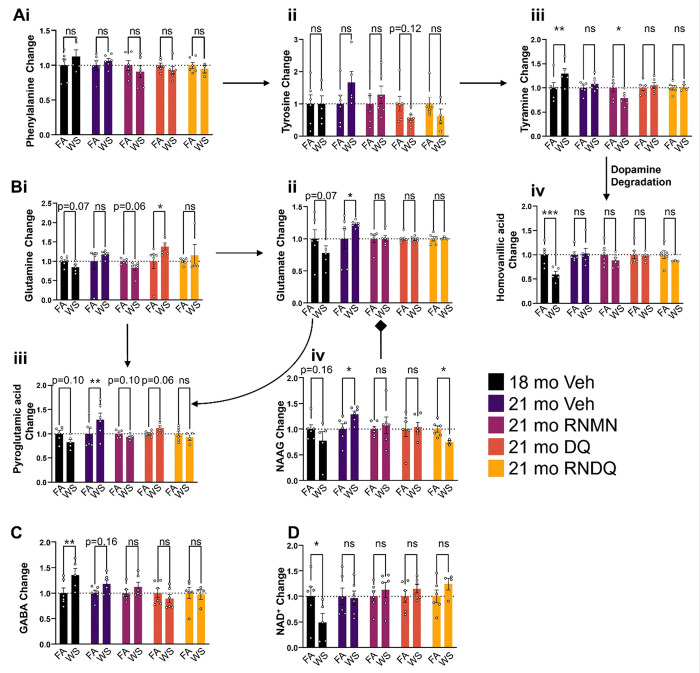
*De novo* neuro-metabolite investigations. Ai-iv) Metabolites detected within the dopamine synthesis and degradation pathway. B i-iv) Metabolites detected in the tripart glutamine, glutamate, and pyroglutamic pathway along with NAAG, which facilitates the release of glutamate. C) GABA D) NAD+. Asterisks denote significant difference from control (*p<0.05; **p<0.01; P<0.001).

A targeted panel of metabolites was used to more specifically query the PFC serotonin and dopamine pathways, as well as N-acetylaspartylglutamic acid (N-acetylaspartylglutamate or NAAG), GABA and NAD^+^. In the dopamine pathway, our untargeted panel was able to detect phenylalanine, tyrosine, tyramine, and homovanillic acid (Fig. 3A i–iv), but dopamine itself was undetected in this analysis. At 1-day post-exposure, 18mo Veh mice saw an upregulation to tyramine (Fig. 3A iii) and downregulation of homovanillic acid (Fig. 3A iv), which are the upstream and downstream metabolites of dopamine, respectively. The tripartite glutamine, glutamate, pyroglutamate pathway (Fig. 3B i–iii) illustrates an increase to glutamate at the 21mo time point. NAAG facilitates the release of glutamate (Fig. 3B iv) and was examined as a potential cause for upregulation of glutamate in 21mo Veh. From these 4 panels, the 18mo Veh glutamate downward trends cannot be explained by other metabolites in this tripartite pathway. However, these data suggest that NAAG is partially responsible for the increases to glutamate and pyroglutamic acid in 21mo Veh. GABA was upregulated 1-day post exposure in the 18mo Veh animals, and we observed trending increases 10 weeks later in the 21mo Veh animals (Fig. 3C). NAD^+^ was decreased immediately after WS exposure, which indicates the potential for accelerated neurological aging (Fig. 3D). Taken together, these data reveal long-term effects of WS on the levels of serotonin, glutamate, NAAG, and GABA (10 weeks), many of which are able to be resolved through the combinations of RNMN, DQ, or RNDQ.

**Figure 4:**
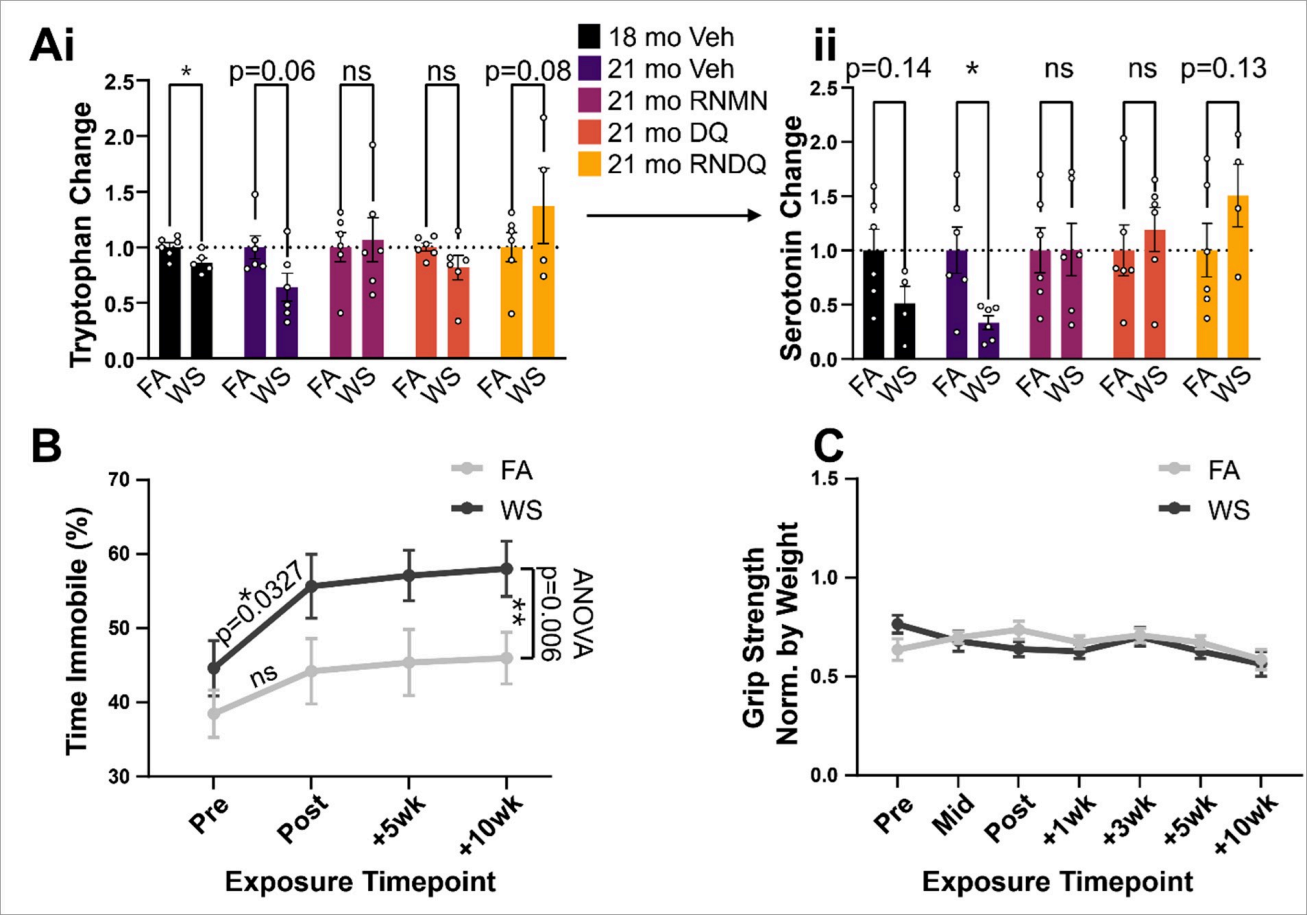
Metabolites and forced swim test measurements. A i & ii) Metabolites detected in the serotonin synthesis pathway. i) Upstream tryptophan. ii) Serotonin abundance. B) Forced swim test. Two-way ANOVA test comparison for FA vs WS over time was conducted. C) Grip. Asterisks denote significant difference from control (*p<0.05; **p<0.01).

We observed significant reductions in serotonin precursor, tryptophan (Fig. 4Ai) at the 18mo time point, which was trending 10 weeks later in the 21mo Veh. These effects were even more prominent in serotonin itself, with a trending reduction immediately post-exposure in the 18mo Veh and a significant decrease 10 weeks later in 21mo Veh animals (Fig. 4Aii). These data indicate a long-term downregulation of serotonin that is unable to be recovered naturally 10 weeks post exposure.

### Validation and Further Assessment of Persistent Neurological Effects

To follow up and validate persistent effects seen 10 weeks after wood smoke exposure, an additional group of female, 18-month-old mice was exposed to WS (N = 8) or FA (N + 8) in a similar regimen and concentration. These mice underwent assessments for behavioral changes (forced swim and grip strength test) and alterations to neuroinflammatory markers.

To further examine the functional outcomes of observed serotonin reductions, we performed forced swim tests 1 day before exposures, 1 day after the last exposure, and after the 10-week recovery period (Fig. 4B). When examining the pre-exposure timepoint, WS-exposed mice showed no significant differences in mobility when compared to FA mice. However, in all post-exposure cases — immediately after exposure, + 5 weeks, and + 10 weeks — immobility was observed as significantly higher in the WS group compared to FA controls, paralleling the sustained decreases in serotonin. Finally, we observed no difference in grip strength, illustrating that these observed effects were not the result of sarcopenia (Fig. 4C). Taken together, these data provide phenotypic evidence that the PFC serotonin changes translated to depression-like behavior that persisted long after cessation of exposures.

**Figure 5:**
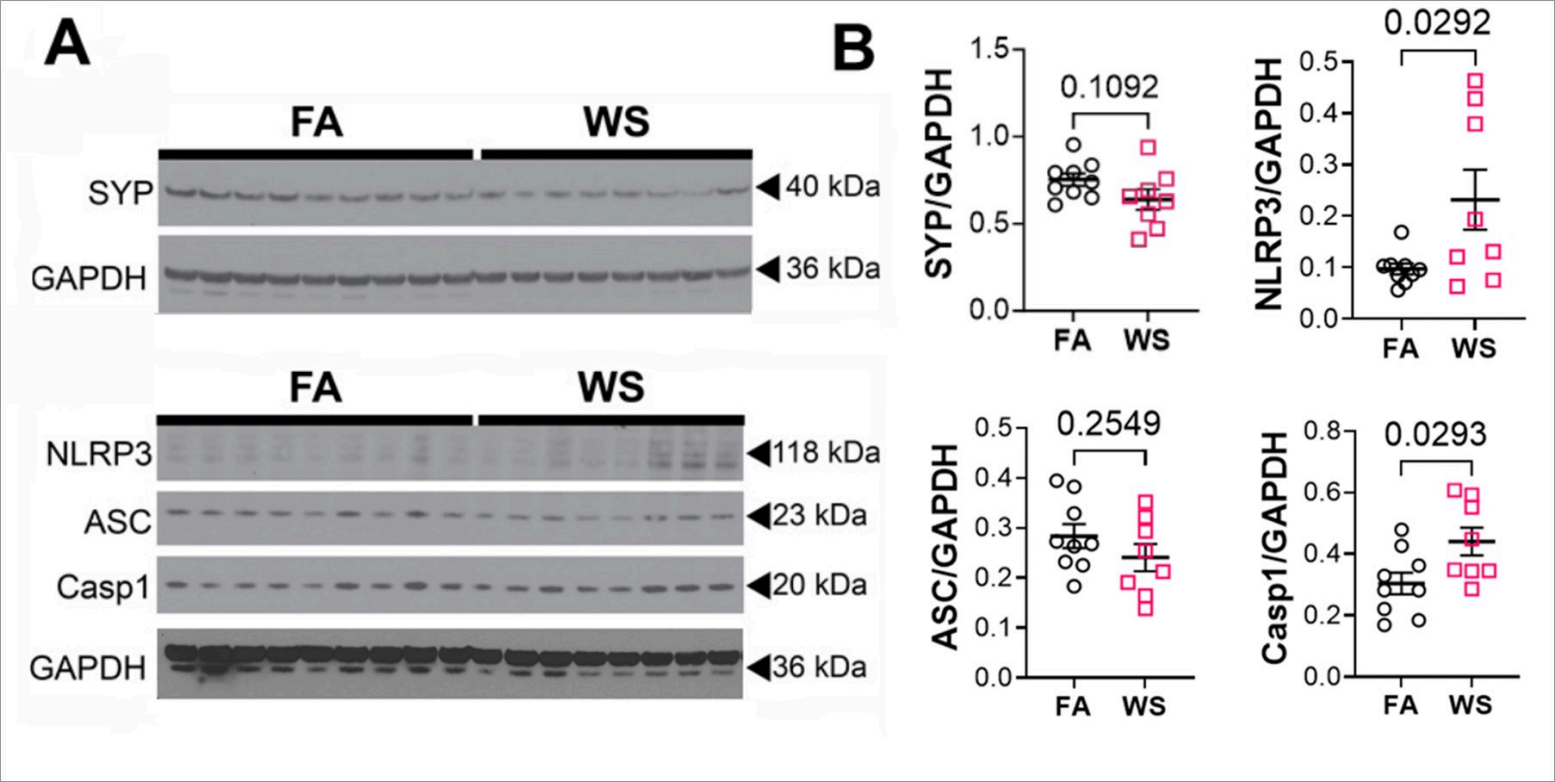
Sustained protein changes 70-days following exposure. A) Western blot of hippocampal lysates for synaptophysin, NLRP3, ASC, and Casp1. B) Quantifications of Western blot densitometry (comparison based on Student’s t-test).

Western blotting of hippocampal lysates was performed immunoblotting for synaptophysin to assess persistent neuronal structural changes that may explain the persistent biochemical and behavioral changes. A modest but not statistically significant reduction in synaptophysin levels was observed in the WS group compared to controls at 10-weeks post-exposure (Fig. 5A). These findings may be explained by the observed reduction in serotonin levels, potentially suggesting reduced pre-synaptic serotonergic inputs in the hippocampus from the median and dorsal raphe nuclei.[58]

Western blotting of hippocampal lysates was also conducted to assess persistent changes in NLRP3 inflammasome levels, key neuroinflammatory proteins associated with long-term neurodegenerative disease.[59–61] 10 weeks after exposures, nucleotide-binding oligomerization domain, leucine-rich repeat and pyrin domain-containing protein 3 (NLRP3) and caspase-1 (Casp1) were both significantly elevated in WS mice compared to control, while apoptosis-associated speck-like protein containing a caspase-activation and recruitment domain (ASC) was unaltered (Fig. 5B). Multiplex ELISA using a pro-inflammatory panel of 10-cytokines was performed to assess persistent changes in mature cytokine levels. Of the cytokines assessed (IFN-γ, IL-1β, IL-2, IL-4, IL-5, IL-6, IL-10, IL-12, KC/GRO, and TNFα), none were significantly altered in WS mice compared to control at 10 weeks (Supplemental Fig. 5). Together, this data indicates that there is persistent elevation in inflammasome priming, but lack of overt cytokine maturation and release in the brain at 10 weeks post-exposure. Such persistent priming of the inflammasome may predispose the animals to increased reactivity to inflammatory stimuli, although further investigation is needed to confirm this outcome.

### Metabolomic similarities between WS exposure and Aging

Finally, a comparison of metabolomic effects between aging and wood smoke exposure was performed (Supplemental Fig. 6A). A total of 129 significantly altered pathways were subset into those related to aging (51), with everything else listed as general (78, Supplemental Fig. 6B). From these, a staggering 70.5% overlapped between aging and exposure (91 pathways, both general and age-related). When subset, 53 general pathways were shared between aging and exposure, and 38 age-related pathways were shared. The effect of aging perturbed 20 general pathways not experienced in the exposure group, and 5 general pathways not experienced by the aging group. Similarly, the aging effect resulted in 9 additional age-related pathways not experienced by the exposure group. However, the exposure group experienced a perturbation of 4 pathways not experienced by the aging group. Overall, our data shows that the effects of aging between 3 months to 21 months has a larger effect both in age-related and general pathways. However, the effects of exposure are not too dissimilar in terms of total pathways altered, as well as age-related and general pathways perturbed.

Two key pathways were noted in common for both exposure and aging that aligned with the sustained behavioral and protein marker changes, namely “long-term depression” and “longevity”. The metabolites for long-term depression impacted by woodsmoke included GLRA4, LDHA, EPRS, MLNR, F2RL3, NGF, SLC29A3, PEMT, LCT, and ALDH2, while the long-term depression metabolites impacted by aging included GLRA4, LDHA, EPRS, P2RY10, FFAR1, NGF, NTHL1, ADORA2B, VIP, CDS1, CKM, PRDM2. The metabolites for longevity regulating pathway metabolites impacted by woodsmoke included MPO, IL1B, CAD, GLUD2, FFAR1, GRIK5, SLC1A4, GOT1, BDKRB1, MLNR, KISS1R, and F2RL3, while the longevity metabolites impacted by aging included MPO, IL1B, CAD, GLUD2, FFAR1, UROD, EEF1E1, AKT1, GNRH1, P2RY10, PROKR2, FFAR2, and ADORA2B. Supplemental Fig. 6C illustrates the overlap in these metabolites for each pathway.

## DISCUSSION

This study examined the immediate and long-term neurometabolomic and neuroinflammatory effects after WS exposure in a murine model of advanced aging, which revealed sustained reductions in PFC serotonin, elevated NLRP3 and Casp1 levels, and lasting behavioral changes up to 10 weeks after cessation of exposure. While the neurometabolomic results largely concur with recent studies in younger, 3-month-old animal models[17], the findings in older 18-month-old animals were more robust and persistent. Importantly, however, resveratrol + NMN treatment was most beneficial after WS exposure compared to exposure-matched, untreated controls, suggesting that there are nutritional/dietary opportunities to offset the detrimental impacts of environmental stressors in advanced age, as an actionable public health approach for an exposure of increasing global concern.

We examined WS because the total acres burned per year in the United States have roughly doubled over the past two decades[62]. PM_2.5_ from wildfires is now negating positive air quality trends in numerous regions, especially in the western United States.[1] The concentrations of WS used in the present study are similar to earlier studies of naturally-occurring wildfire PM[16, 17], and well within range of human exposures that occur routinely during summer months[63]. Thus, we were specifically modeling an exposure concentration that millions of individuals may experience within a few hundred miles of the wildfire source, but sufficiently distant that evacuation would not be considered. Notably, our laboratory-based exposure system does not recapitulate the atmospheric aging associated with long-range transport, but the neuroinflammatory outcomes were quite similar between laboratory-generated and naturally occurring smoke exposures [16, 17]. Although many of the toxic gaseous components dissipate within close proximity to the fire source, the stability of carbon monoxide and PM_2.5_ lead to transport over great distances in the air; PM_2.5_ from wildfire smoke drove neurological outcomes thousands of kilometers away from the fire origins[16].

The study was designed to investigate how WS exposure might alter the trajectory of natural aging, utilizing a window from 18 to 21 months of age when many physiological systems are in decline in mice. The selection of animal age was largely based on the increased susceptibility of older animals to environmental insults and exposures [64, 65]. Previously, data from our lab showed that 3-month-old female mice were unable to completely resolve woodsmoke effects within 30 days of final exposure [17] (37608305). In the present study, we opted for a model of enhanced aging at 18–21 months of age to see whether this neuroinflammatory insult would prime a more rapid decline. Expanding upon the most striking findings in aged mice, WS exposure caused a sustained reduction in serotonin levels in the prefrontal cortex that persisted even after 10 weeks of recovery. The phenotypic expression of this change was supported by increased immobility in the forced swim test, suggesting the serotonin changes may have translated to increased depression-like behavior. These results parallel human studies showing a decreased ability to focus and learn after wildfire smoke exposure[15]. Numerous epidemiological studies support a link between PM and neurological and behavioral health[6, 66, 67], but few have addressed wildfire smoke, specifically. Neurological and psychiatric outcomes associated with PM_2.5_ exposure include depression, anxiety, suicide, ADRD, and decreased learning ability, in addition to cardiopulmonary outcomes that can be fatal[6, 11–13, 15, 68–70]. A recent study, however, observed that risk of dementia from wildfire-derived PM was positive along with dusts from agricultural sources, while most other sources of PM were not associated with dementia outcomes[14]. Our findings show that mice exhibited a depressive-like phenotype of reduced mobile time in a swim test after woodsmoke exposure and this was not due to sarcopenia per the grip test. However, cardiorespiratory deficits may be an important outcome of WS exposure and could contribute to to the observed behavioral changes.

Based on our experimental design (Fig. 1A), we were able to query neurological effects of wildfire smoke and natural aging through metabolomic mass spectrometry. Previous work from our lab[16] and others[15, 71, 72] revealed metabolite alterations that were suggestive of mood alterations and impaired memory formation following WS exposure. Thus, we dissected the PFC to determine these effects and modeled them against aging, natural resolution of response, and the comparison of each drug regimen. To further assist in determining age-related effects, we employed a metabolomic panel designed to investigate the NAD^+^ synthesis pathway, and an additional untargeted panel. This experimental design was specifically chosen because it afforded the ability to examine a multitude of effects. Namely, the immediate exposure effects were determined through a comparison of FA vs WS at terminus 1 (1-day post final exposure).

We could determine natural resolution of inflammation over 10 weeks after therapeutic dosing (dark purple). Our results showed that neuroinflammation was largely resolved after 10 weeks post-exposure as indicated by unaltered inflammatory cytokine levels compared to FA mice. However, NLRP3 inflammasome protein levels remained significantly elevated indicative of persistent inflammasome priming in WS mice [73]. The inflammasome plays an important role in inflammaging, a chronic sterile inflammatory process in ageing that contributes to increased morbidity and mortality[74–76]. Our findings suggest that WS may serve as an environmental trigger mediating the inflammaging process. Through the combinations of different drug regimens, we could compare and contrast a multitude of effects, like natural aging, WS-associated aging, and drug rescuing effects, among others. For example, the difference between natural aging can be examined by comparing FA 18-month-old (18 mo) vehicle (Veh) to FA 21-month-old (21 mo) Veh. These naturally altered age-associated metabolites can be compared to those found from WS 18 mo Veh vs Ws 21 mo Veh for overlap. Additionally, we cannot rule out the possibility of epigenetic alterations following exposure. The rate of epigenetic aging (biological age) has been measured against the chronological age as a marker of accelerated or decelerated aging. Although not investigated in this study, it would be worthwhile to determine the effects of wildfires and woodsmoke on the rate of neurological aging as this could shed light on the current state of unknown health risks.

### Limitations and Summary

Several limitations for the study should be noted. As resveratrol activates NAD^+^ consuming enzymes for longevity-associated effects and NMN is a precursor to the enzymatic fuel source, NAD^+^, this combination was viewed strategically for the aging mouse model, but we did not assess the specific impact of either resveratrol or NMN in isolation. The choice of biomass (pinon wood) for this study was based on local fuel sources and may differ from the numerous other sources. Importantly, wildfires are uncontrolled mixtures of grasses, shrubs, and trees, burning at different temperatures, with meteorological factors influencing transport, mixing, and atmospheric aging of PM. Thus, there may not be a perfect model for wildfire smoke exposure, given the variations of time, distance, and concentration to which millions of people are exposed. We specifically examined the PFC for metabolomic data and made no comparisons to other regions of the brain. While we anticipate that neuroinflammation from wood smoke exposures is widespread in the brain, different regions may respond differently than what was observed in the PFC. Similarly, we specifically examined the hippocampus for inflammasome proteins, inflammatory cytokines, and synaptophysin levels but did not assess other brain regions. Additionally, inflammatory cytokine and inflammasome levels were only assessed 10 weeks post-exposure to assess any persistent changes. We were unable to assess whether neuroinflammation increases acutely after WS exposure in this aged model, although, we have previously reported invasion of peripheral immune cells and reactive microglia within days to weeks after WS exposure in young 8-week-old mice.[17] Lastly, the current study only examined female mice and is not generalizable to both sexes of mice without investigation.

Summarily, these data reveal persistent alterations in the neurometabolome of aging mice following exposure to WS, with corroborating behavioral impacts and persistent inflammasome priming. The WS exposure tended to promote or exacerbate the metabolomic changes associated with aging, suggesting that not only is aging a risk factor that promotes vulnerability to environmental contaminants, but that environmental contaminants may drive certain processes of aging. Findings help explain the epidemiologically-observed associations between PM exposure and neurological outcomes, and further highlight the need to better understand the neurological and psychological impacts that wildfire smoke may have on public health, and practical interventions to offset those effects.

## MATERIALS AND METHODS

### Animals and Wood Smoke Exposures

Female C57BL/6J mice (Jackson Labs) at 18 months of age were housed in AAALAC-approved facilities, on a 12h light:dark (7AM:7PM) cycle and allowed to acclimate for 1 week prior to experiments. Depending on experimental condition, they were provided either standard chow diet or resveratrol chow (0.1% w/w, Teklad Envigo) and DI water or nicotinamide mononucleotide (NMN, Millipore Sigma) water (described below) ad libitum (Fig. 1A). A total of 60 mice were used, randomly and evenly divided into filtered air (FA) control and WS (WS) exposed groups; with 6 mice per reusable plastic animal case system (Fig. 1A). All procedures were conducted humanely with approval by the University of New Mexico Institutional Animal Care and Use Committee. For exposures, a BioSpherix Medium A-Chamber was used, with mice in reusable shoebox plastic animal case systems. They were outfitted with standard wire tops, and water was available to mice throughout the exposures, but food was withheld (4h/d).

Whole-body exposures to biomass combustion were conducted for 4 h every other day for 14 days (7 total exposures). Biomass smoke production was facilitated by a ceramic furnace encircling a quartz tube using chipped pinon wood as the fuel[17]. This tube was connected to a dilution chamber, with subsequent plumbing into the exposure chamber (Supplemental Fig. 1). Exposure chamber smoke abundance was facilitated by vacuum and/or pressurization, with total pressure monitoring to ensure min/max exposure chamber pressure never exceeded −/+ 25mm Hg. Concentrations were monitored in real-time and manually adjusted to provide consistent average exposure levels.

Exposure concentrations were measured with a with a DustTrak II (TSI, Inc; Shoreview, Minnesota) in real-time using a tube fed directly into the exposure chamber. 47 mm quartz filters were collected for the duration of each exposure, and gravimetrically confirmed final daily averages using a microbalance (XPR6UD5, Mettler Toledo) in a temperature-controlled laboratory. Particle size distribution was quantified using the TSI Laser Aerosol Spectrometer 3340A using a tube fed directly to the exposure chamber. Size distribution was acquired without mice in the exposure chamber, and distribution was measured over a single 2-hour run. Particle size distribution largely fell within PM _0–1_ (median range = 0.138–0.145 μm) with less than 1% of particles above PM_2.5_.

### Post-exposure Recovery and Drug Treatments

After the last round of exposures, one group of mice were euthanized (6 FA and 5 WS-exposed mice) with the remaining 24 mice per exposure group then randomized to 1 of the 4 drug regimens (Fig. 1A): (1) standard chow, deionized (DI) water, vehicle (Veh) gavage; (2) resveratrol milled into standard chow, NMN water, Veh gavage (RNMN); (3) standard chow, DI water, dasatinib and quercetin (DQ) gavage; (4) resveratrol chow, NMN water, DQ gavage (RNDQ). An additional group of mice was included to recapitulate metabolomic findings of group 1 (Veh control) in addition to applying a forced swim test and grip strength test to characterize potential behavioral implications. Resveratrol chow was achieved by milling 0.1% resveratrol by weight into standard chow (Envigo, WI, USA). Estimated intake of resveratrol was calculated as 5mg/30g mouse/day, or 167 mg/kg/day[77]. NMN was added to water 1.3mg/mL per week. Estimated intake of NMN was calculated as 300mg/kg/day[78]. Mice were gavaged with the formulation of Dasatinib (5 mg/kg, Thermo) and Quercetin (10 mg/kg, Thermo) in vehicle (60% Phosal, 10% ethanol, 30% PEG-400) or gavaged with vehicle alone; ethanol and PEG-400 Phosal formulation was 50% phosphatidyl choline and 50% propylene glycol (Thermo). Dasatinib and quercetin gavage stock was created the day of each administration. Drugs or vehicle were administered at 9–11am for 3 consecutive days per week (i.e., Monday-Wednesday), every other week, for the total period of 10 weeks[79].

### Forced Swim and Grip Strength Testing

A separate subset of mice underwent exposures to FA (N = 10) or WS (N = 10) as above and then administered a forced swim test and grip strength test (Supplemental Fig. 2, Round 2). 1 day before and 1 day after the 14d exposure period, mice were placed into clear cylindrical plastic buckets measuring 24 cm in height and 19 cm in diameter, which were filled with 23–25°C tap water to a depth of 16–20 cm. Swim sessions were recorded over the course of a 6-minute test, last 4 minutes scored, using a camera mounted on a tripod, which was positioned so its lens was level with the water surface in the bucket. For the pre-exposure test, an observer, unaware of the treatment conditions, analyzed the animals’ behavior. A time sampling technique was employed to categorize the predominant behavior as swimming, immobility, or climbing. Additionally, behavior was scored automatically using DBscorerV2. Manual observations by the observer were scored every 5 seconds during the final 4 minutes of the test. Meanwhile, the DBscorerV2 scoring followed best practice guidelines provided in the original mauscript[80]. Briefly, videos were loaded, start and endpoints were selected, clean backgrounds were created without mice, regions of interest were marked, and auto-subject selection was performed. When video artifacts existed, background selection was cleaned, and delta area threshold was set at 2.6%. Comparisons between observer and DBscorer evaluations revealed no significant discrepancies. Consequently, DBscorerV2 was used for all subsequent swim tests.

A force transducer (Series 2 Mark-10; JLW Instruments, USA) was placed vertically and used to measure forelimb grip strength[81]. Peak tensions were measured between 8AM – 11AM each testing day. Replicates were performed 3 times mouse with 1-minute breaks between. Replicates were averaged and normalized by mouse weight taken on the same morning[82]. Procedurally, mice were allowed to grip the bar and the tail was slowly pulled, allowing mice to build up resistance to the force applied. In each replicate, trials resulting from a single paw grip, hind leg assistance, or a mouse body angle < 30° off center relative to the tensometer were excluded and repeated.

### Brain Tissue Dissection

Under isoflurane anesthesia, all mice underwent transcardial ice-cold 0.1M PBS (pH = 7.4) perfusion and subsequent steps were performed quickly. Surgical scissors were used to cut skulls from caudal to rostral along the medial edge until reaching the frontal bone anterior to bregma. Skulls were reflected rostrally to expose intact brains and olfactory bulbs. Brains were removed using a sterilized spatula and placed onto ice-cold watch glass with a sterile filter paper cover that had been soaked in ice-cold PBS (minus calcium and magnesium). Brains were hemi-sected with a sterile razor blade. Each left PFC was excised using a sterile razor blade and placed into a cryovial before plunging into liquid N_2_ for rapid freezing. Right hippocampi were micro-dissected using sterile fine forceps, wet-weights were recorded after placing into a cryovial, and then the tissue was rapidly frozen on dry ice and stored at −80°C for biochemical analysis.

### Metabolomic Tissue Preparation and Analysis

Each PFC sample (~ 20 mg, n = 6) was homogenized in an Eppendorf tube using a Bullet Blender homogenizer (Next Advance, Averill Park, NY) in 200 μL methanol:PBS (4:1, v:v, containing 1,810.5 μM 13C3-lactate and 142 μM 13C5-glutamic acid) with a final addition of 800 μL methanol:PBS (same v:v). Samples were vortexed again for 10 s, stored at −20°C for 30 min and sonicated in an ice bath for 30 min. After a centrifugation step at 14,000 RPM for 10 min (4°C), 800 μL of supernatant was transferred to a new Eppendorf tube. The samples were dried under vacuum (CentriVap Concentrator, Labconco, Fort Scott, KS). The obtained residues were reconstituted in 150 μL 40% PBS/60% acetonitrile. A quality control sample was pooled from all the study samples.

### Liquid Chromatography–Tandem Mass Spectrometry (LC–MS/MS)

Targeted and untargeted LC-MS/MS techniques were similar to several recent reports[83, 84] using an Agilent 1290 UPLC-6490 QQQ-MS (Santa Clara, CA) system. Each PFC sample was injected twice: (1) 10 μL volume for negative ionization mode analysis and (2) 4 μL volume for positive ionization mode analysis. Both chromatographic separations were performed in hydrophilic interaction chromatography mode on a Waters XBridge BEH Amide column (150 × 2.1 mm, 2.5 μm particle size, Waters Corporation, Milford, MA). A flow rate of 0.3 mL/min, auto-sampler temperature of 4°C, and a column compartment temperature of 40°C were employed. The mobile phase was composed of Solvents A (10 mM ammonium acetate, 10 mM ammonium hydroxide in 95% H_2_O/5% acetonitrile) and B (10 mM ammonium acetate, 10 mM ammonium hydroxide in 95% acetonitrile/5% H_2_O). Initial 1 min isocratic elution of 90% B, decreased 40% B for 4 minutes (at t = 11 min till t = 15 min). The percentage of B gradually went back to 90%, to prepare for the next injection. The mass spectrometer is equipped with an electrospray ionization source. Untargeted data acquisition was performed in multiple-reaction-monitoring mode. The whole LC-MS system was controlled by Agilent Masshunter Workstation software (Santa Clara, CA). The extracted MRM peaks were integrated using Agilent MassHunter Quantitative Data Analysis (Santa Clara, CA). Metabolites were obtained by querying against 4 separate databases, including mzCloud, Metabolika, ChemSpider, and MassList. Final metabolites were selected based on a cutoff CV(QC) < 20% and an ID score of 1, where 1 is equivalent to a full match on predicted compositions, full match between all databases queried, and DDA for preferred ion. LC-MS grade acetonitrile, methanol, ammonium acetate, and acetic acid were purchased from Fisher Scientific (Pittsburgh, PA). Ammonium hydroxide was bought from Sigma-Aldrich (Saint Louis, MO). Standard compounds were purchased from Sigma-Aldrich and Fisher Scientific. Resultant data were normalized by tissue weight before subsequent normalization steps.

### Western Blot and ELISA Tissue Preparation and Analysis

Each hippocampal sample (~ 25 mg, 10% weight/vol) was homogenized for 1 min on ice in an Eppendorf tube in Tissue Protein Extraction Reagent (TPER^®^, Thermo Scientific, Catalog #78510) with phosphatase and protease inhibitor cocktails (Sigma Aldrich, P5726 and P8340, respectively) and sonicated with a probe-tip sonicator for 30 s at 20% A on ice. Soluble hippocampal lysates were centrifuged at 12,000 g for 30 min at 4°C and aliquoted for ELISA or Western blot analysis.

Western blot samples were boiled for 15 min at 95°C in NuPAGE^™^ LDS and RA (Invitrogen^™^, NP0007 and NP0004, respectively). Samples were resolved on NuPAGE^™^ 4–12% Bis-Tris gels (Invitrogen) and immunoblotted overnight on 0.45μM PVDF membranes (Thermo Scientific). Primary antibody dilutions were as follows: synaptophysin (Sigma, catalog # SAB4502906) 1:5000, NLRP3 (AdipoGen, Cryo-2) 1:2,000, ASC (AdipoGen AL177) 1:2500, caspase-1 (Santa Cruz, SC-398715) 1:1000, GAPDH (Millipore, AB2302) 1:10,000. HRP-conjugated secondary antibodies raised against their respective host species (goat-anti-mouse: Jackson ImmunoResearch, catalog # 115-035-003; goat-anti-rabbit: Jackson ImmunoResearch, catalog # 111-035-003) were consistently diluted at 1:10,000. Pierce ECL Western blotting substrate (Thermo Scientific, catalog # 32109) was used to generate a chemiluminescent signal and immediately developed on radiography film. Scanned western blot films were analyzed using AlphaEaseFC^™^ v.3.2.1 and statistical analysis was performed using GraphPad Prism v.10.2.3.

Hippocampal lysates were also assessed for inflammatory cytokine levels using the Meso Scale Discovery© V-Plex Pro-Inflammatory Panel 1 Mouse Kit (MSD, K15048D-1). The assay was performed according to the manufacturer’s instructions using 50 μL of undiluted hippocampal lysates (10% w/v) in duplicate. The average concentration of inflammatory cytokine detected in each sample was then normalized to a total protein concentration of 100 μg for each sample determined by BCA assay (Thermo Scientific, catalog #23225).

### Data Analysis and Statistics

The quality control samples (inserted at 5-sample intervals during mass spectrometry) were utilized as a pooled sample group to compensate for temporal variability on the machine. Metabolomic data was analyzed using the R package MetaboAnalystR. Normality was determined via Shapiro-Wilk testing. In the event of normally distributed data, student’s t-tests were used. For non-normally distributed data, t-tests were employed on log_2_() or log_10_() transformed data. Tests were either conducted in GraphPad Prism v10.2.3, Excel (Version 2211 Build 16.0.15831.20098) or Rstudio v1.4.1564. Venn diagram was generated using an online multiple list comparison tool[85]. For Venn diagrams, uncorrected student’s t-tests were performed as these data were not individually examined, but qualitatively explored as overlapping matrices. These values were input into excel, Rstudio, or downloaded directly for figure generation. Volcano plots were generated using an FDR p < 0.1 and fold-change threshold > 1.5. Linear mixed effects regression modeling was performed with the packages limma, lme4, lmerTest, emmeans, multcomp, and lsmeans. Data were analyzed with fixed effects being age, exposure, and drug combination, with each metabolite being nested, and sample number called as a random effect. Results were holm-corrected before being plotted with the ggplot2, dplyr, and tidyr packages. Behavioral and other data were analyzed in GraphPad Prism using two-way ANOVAs or Student’s t-tests. Pathway analysis was performed using the metaboanalyst network analysis tool. Statistically significant metabolites with fold-changes were aggregated from linear mixed effects regression (described above) and metabolites without direct match/hits within the tool were disregarded. Both the aging and exposure metabolites were input using a degree filter of 2.0, betweenness filter of 3.0, and minimum network was selected. The exposure effect resulted in 1 subnetwork with 665 nodes, 2696 edges, and 62 seeds. The aging effects also resulted in 1 subnetwork, but with 731 nodes, 3303 edges, and 68 seeds. For each separate condition (aging or exposure), all nodes were used to determine statistically significant KEGG pathways and downloaded for further analysis. Pathways known to be associated with age-related alterations, depression, and those that have a direct effect on regulating longevity were determined through literature search via PubMed and ConsensusAI. When pathways overlapped between comparison conditions, they were color coded (e.g., longevity regulation pathway is statistically significant for aging and for exposure, color code in red). For numerical representation of overlapping pathways, UpSet plots were generated using the UpSetR package.

## Data Availability

All data related to this study are publicly available upon reasonable request to the corresponding authors.

## Abbreviations

ADRD: Alzheimer’s disease related dementias
ANOVA: Analysis of Variance
BCA: Bicinchoninic acid
D+Q: Dasatinib + quercitin
ELISA: Enzyme-linked immunosorbent assay
FA: Filtered air
NAD^+^: Nicotinamide adenine dinucleotide
NMN: Nicotinamide Mononucleotide
PM: Particulate matter
PFC: Pre-frontal cortex
RNMN: Resveratrol plus nicotinamide mononucleotide
WS: Woodsmoke
